# Micromachined optical flow cell for sensitive measurement of droplets in tubing

**DOI:** 10.1007/s10544-018-0337-x

**Published:** 2018-10-29

**Authors:** Sammer-ul Hassan, Adrian M. Nightingale, Xize Niu

**Affiliations:** 1 Faculty of Engineering and the Environment, University of Southampton, Southampton, SO17 1BJ UK; 2 Institute for Life Sciences, University of Southampton, Southampton, SO17 1BJ UK

**Keywords:** Microfluidics, Droplet microfluidics, Absorbance, Real-time monitoring, Continuous measurement, Enzymatic assays, Optical detections, Point-of-care diagnostics

## Abstract

Here a micromachined flow cell with enhanced optical sensitivity is presented that allows high-throughput analysis of microdroplets. As a droplet flows through multiple concatenated measurement points, the rate of enzymatic reaction in the droplet can be fully characterized without stopping the flow. Since there is no cross-talk between the droplets, the flow cell is capable of continuously measuring biochemical assays in a droplet flow and thus is suitable to be used for continuous point-of-care diagnostics monitoring. This paper describes the design and operation of the device and its validation by application to the accurate and continuous quantification of glucose concentrations using an oxidase enzymatic assay. The flow cell forms an important component in the miniaturization of chemical and bio analyzers into portable or wearable devices.

## Introduction

Miniaturization of chemical assays in microfluidic devices has become practical with the development of microfabrication technologies, with the significant advantages such as reduced amounts of samples and reagents, cost-effectiveness, robustness and sensitive assays (Whitesides 2006). In general, these systems are developed to provide high-throughput, parallel, and automated analytical analyses.

Continuous microfluidics normally involves single phase flow in microchannels (Teh et al. 2008). Devices based on continuous microfluidics have been developed to scale down the amount of reagents from millilitres or microlitres to nanolitres and are used to study chemical reactions or synthesize materials in a short interval of time. However, there are a number of disadvantages associated with continuous microfluidics such as slow and weak mixing in microchannels, Taylor dispersion of the analytes and sample loss or contamination of biomolecules on the surface of the channel walls (Teh et al. 2008; Huebner et al. 2008).

In contrast, droplet-based microfluidics encapsulates biomolecules or analytes of interest into discrete droplets and perform analysis with these ‘digital’ units (Sista et al. 2008). In droplet microfluidics, nanolitre to femtolitre sized discrete droplets are generated in the microchannel by pinching off continuous aqueous stream with an immiscible carrier phase, which is usually oil. Mixing in droplets is faster as compared to continuous microflows because of the fluidic circulations inside the droplets (Tiselius 1937). Analytes of interest are compartmentalized into droplet plugs, and the carrier phase prevents the sample from contact with the surface wall and hence, eliminates sample loss on the channel surfaces. Carrier phase also prevents the leakage of the molecules and cross-contamination between the droplets. Thus, in droplet-based microfluidics, each droplet acts as a separate microreactor and multiple of them (as many as millions) can be transported and analysed in a single device (Ostergaard and Jensen 2009). Whereas in continuous microfluidics, it requires separate microchannels for each sample loading or complicated fluidic controls to clean the channels between the sample loadings due to the contamination problems.

Colorimetry, in which an analyte-specific reagent reacts with the target analyte to give a coloured product, offers a promising platform to quantify analytes via absorption spectrophotometry and is highly suitable for accurate quantitative analysis in microfluidics for multiple biochemical assays (Ellerbee et al. 2009; Sieben et al. 2010; Rushworth et al. 2015; Tietz et al. 2006). Colorimetric assays are typically slow (Nelson and Cox 2008; Sugiura and Hirano 1977), therefore, a rate kinetic method is used to quantify the metabolite concentrations. Colorimetric assays have already been applied in droplet microfluidics such as Srinivasan et al. (2004) developed a droplet-based system to measure the kinetics of glucose in droplets. The optical detection was performed on a plane perpendicular to microfluidic chip consisting of LED and photodiode with a path length of 475 μm. Droplets of glucose and reagents were pipetted onto the electrowetting plate, merged to produce a final volume of 2 μL, mixed for 15 s, and the absorbance measurements were taken for the 30s to generate Michaelis-Menten parameters. Similarly, Fradet et al. (2015) fused the droplets of sample and reagent (β-d-glucosidase and 4-nitrophenyl β-d-glucopyranoside), immobilised the resulting droplet and followed the colour development over time using an optical microscope. The authors studied different enzyme concentrations, generated Michaelis-Menten kinetics, and quantified the effect of the inhibitor on enzyme activity. In a recent study, Gielen et al. (2015) developed a sampler that was able to generate high-resolution concentration gradients of haloalkane dehalogenase DbjA and analysed 150 combinations of enzyme and substrate in less than 5 min. In another study Gielen et al. (2013) chose to monitor the similar enzymatic reaction by oscillating reaction-containing droplets back-and-forth through an absorption flow cell. The authors generated droplets of 4-nitrophenyl glucopyranoside hydrolysis by sweet almond β-glucosidase and passed through the detector consisting of LED and photodiode with a path length of 200 μm. Different concentrations of the substrate were loaded into the droplets, merged to initiate the reaction, and the absorbance was measured for each set of concentration by flowing the droplets back and forth in the detector to generate Michaelis-Menten parameters. The above-mentioned methods were able to follow the color development in detail and obtained accurate reaction rates, however, the throughput is still low. The chief reason for such a low throughput analysis is due to a single detection point to measure the color development in the droplets, therefore, either stop flow or oscillation flow is needed. This low throughput analysis has yet to match the potentials of high throughput analysis that droplet-based microfluidics can offer, for example, thousands of droplets can be produced per second in a single T-junction (Gu et al. 2011).

This paper shows how sensitivities can be improved by upgrading the flow cell from 3D printed cartridge (Hassan et al. 2016; Nightingale et al. 2017) to a micromilled cartridge with the limit of detection (LOD) of 0.1 mM as compared to 0.35 mM (3D-printed).

## Materials and methods

### Materials and reagents

Glucose oxidase, horseradish peroxidase, 4-aminoantipyrine, phenol and phosphate buffered saline (PBS) tablets were bought from Sigma-Aldrich (Dorset, U.K) and prepared in 0.1 M PBS at pH of 7.4. Ultrapure water (18.2 MΩ cm, MilliQ) was used for the preparation of all solutions. The reagents were mixed in equal volumes to achieve the final concentrations as follows: glucose oxidase (30 U mL^−1^), peroxidase (30 U mL^−1^), 4-aminoantipyrine (1.54 mM) and phenol (22 mM). Red food dye (East End Foods plc) and D-glucose were dissolved in the DI water and 0.1 M PBS respectively to prepare the standard solutions.

### Droplet generation

T-junction microchip to generate droplets was fabricated by designing a mould in SolidWorks (Dassault Systemes), printing (Objet500 Connex3 3D printer, Stanford Marsh Ltd) and half curing PDMS bonding as outlined previously (Hassan et al. 2016). One inlet was used to pump FC-40 carrier fluid with 0.35% *w*/w (surfactant non-ionic tri-block copolymer surfactant) synthesized in-house (Utada et al. 2005). The sample and the reagents were injected in a volume ratio of 1:1 via Syringe pumps (PHD 2000/Harvard Apparatus). Polytetrafluoroethylene (PTFE) tubing (0.4 mm ID, 0.7 mm OD, Adtech Polymer Engineering Ltd.) was used to transport the droplets through the flow cell.

### Flow cell fabrication

**Fig. 1:**
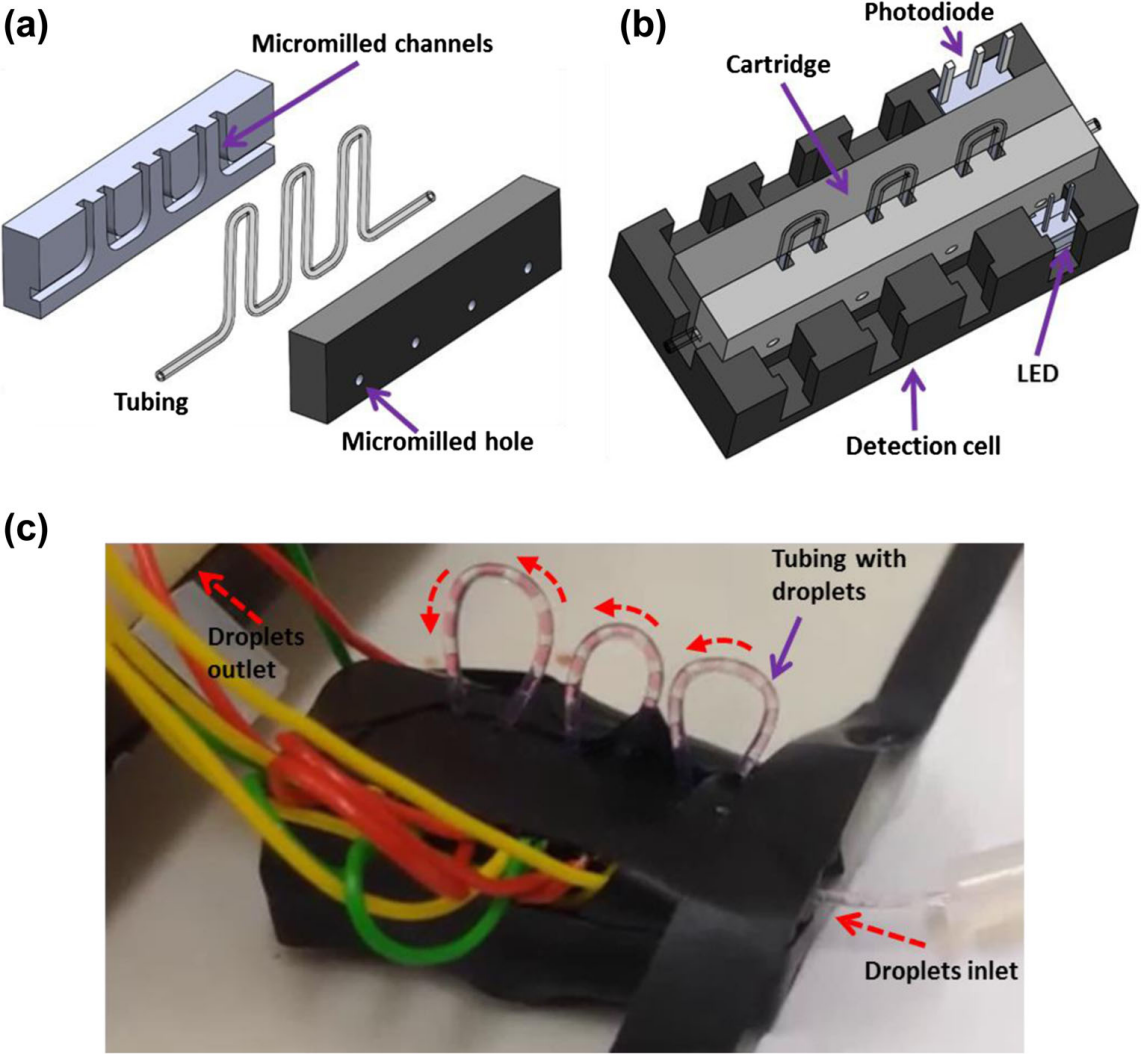
Multi-detector flow cell: **a** 3D schematic of the micromilled cartridge showing two PMMA parts with holes, channels, and tubing; **b** 3D schematic of the fully assembled 4-detector flow cell illustrating cartridge, tubing, LED and photodiode; **c** Snapshot of the detector showing cartridge in place and tubing with droplets flowing through the flow cell

The flow cell was designed with SolidWorks and AutoCAD as shown in a 3D schematic in Fig. 1. It consists of two parts i.e. detection cell and the cartridge. Detection cell was fabricated via 3D printing in black poly(lactic acid) (PLA) material as described previously (Hassan et al. 2016). However, the cartridge is fabricated via micromilling of the polymethylmethacrylate (PMMA) pieces contrary to the 3D printed cartridges previously fabricated (Hassan et al. 2016). This is because the precise micromilling allows excellent alignment of the holes and channels and reduces the variation in absorbance values introduced due to the variability in slit sized (detector-to-detector variability reduced (%RSD) to 3.3% from 6.5%).

As indicated in the schematic of the cartridge in Fig. 1a, there are 4 opening holes in the channels that provide the passage for light. The holes (0.4 mm) and a channel (0.7 mm) to hold the tubing were micromilled in a PMMA piece (3 mm thick) using LPKF Protomat S100 micromilling machine as discussed previously (Hassan et al. 2015, 2017). The second part was also fabricated similarly without the tubing channel (Fig. 1a). Both parts were aligned via alignment pins and glued ((Super Glue, Loctite) together. The cartridge can slot into the detection cell (Fig. 1b). The detection cell was equipped with LEDs (ASMT-QGBE-NFH0E, Avago Technologies) and light to voltage convertors (TSL257, Texas Advance Optical Solutions). Figure 1c shows the schematic of the fully assembled 4-detector flow cell (the size of the device is 28 × 8 × 6 mm).

### Data analysis

A microcontroller (Arduino Nano) was used to collect the signal from the photodiode which was displayed via LabView (National Instruments) connected to the computer. Beer-Lambert law was modified to measure the absorption for each droplet which normalises the intensity values based on the intensity values of FC-40 oil levels (Hassan et al. 2016).

## Results and discussion

**Fig. 2:**
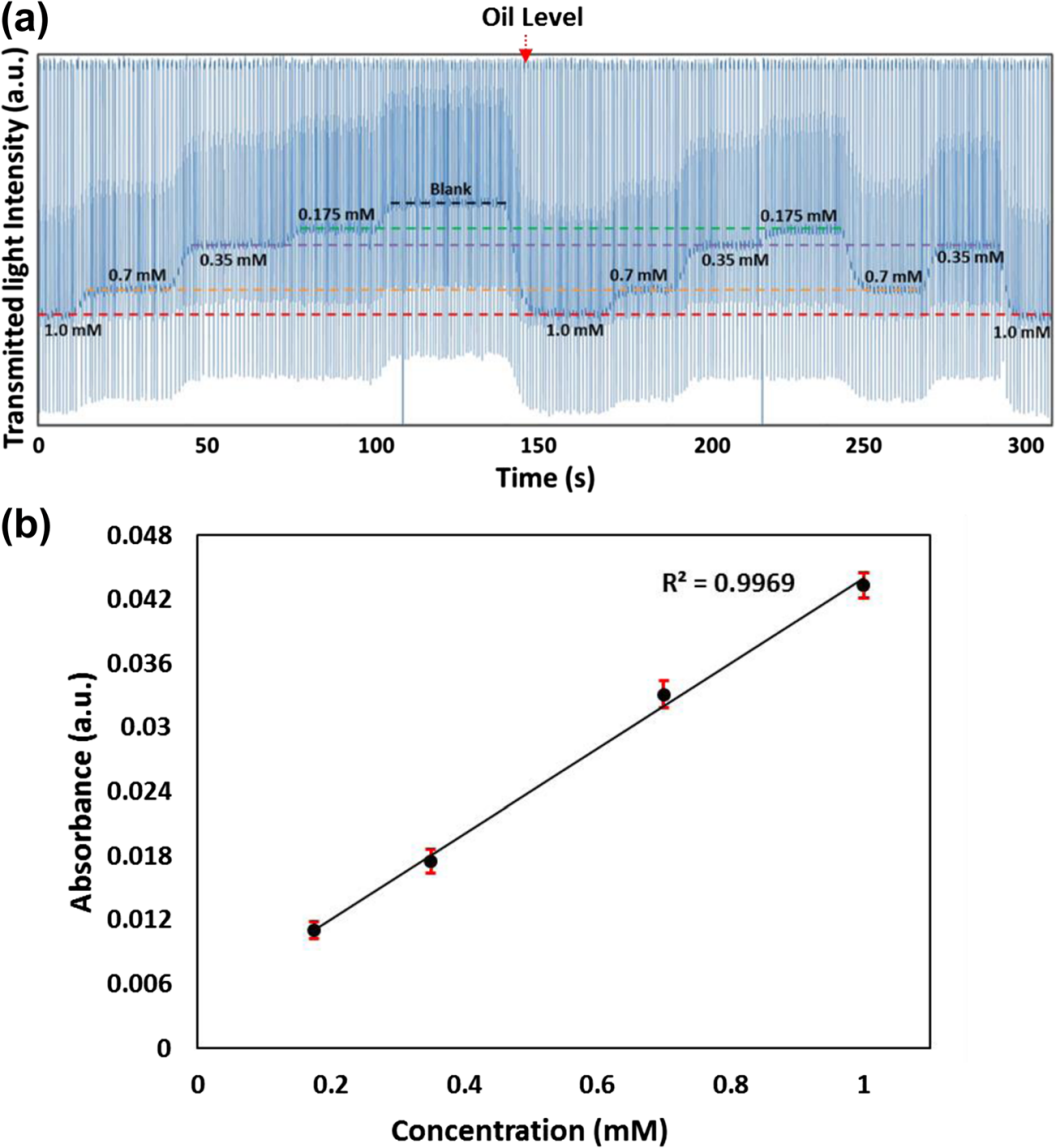
Rapid glucose change detection in the flow cell: **a** Glucose concentrations of 0.175, 0.35, 0.7 and 1.00 mM were injected into the microchip randomly, and the transmitted light intensity signal was recorded. The concentrations were changed by switching the glucose solution inlet tubing without stopping the pump. The dotted lines indicate the intensity levels of droplets for each specific concentration after switching; **b** The linear best fit of absorbance values versus concentration shows that the change in absorbance is linear for all concentrations at a detection time of 21.6 s

The sensitivity of the flow cell was tested by generating droplets of glucose reaction mixture (colorimetric glucose enzymatic assay via Trinder’s assay) (Hassan et al. 2016) and injecting them into the single detector flow cell. One inlet was pumped with reagent mix and the other inlet was pumped with glucose concentrations (0.175, 0.35, 0.7, and 1.0 mM) at room temperature. Blank measurements were also taken by injecting blank in one inlet and reagents in another inlet. The concentrations were switched randomly by switching the inlet tubing of the glucose solutions to observe the sensitivity of the detector towards lower glucose values. Droplets travelled through the tubing and entered the flow cell at a time of 21.6 s. The transmitted light intensity was recorded as shown in Fig. 2a which clearly shows that a small change in the glucose concentration can be quantified with a micromilled version of the cartridge as compared to the 3D printed cartridge. Figure 2b shows calibration curve for absorbance versus concentrations and the absorbance values fit very well with the linear best fit (R^2^ = 0.9969).

**Fig. 3:**
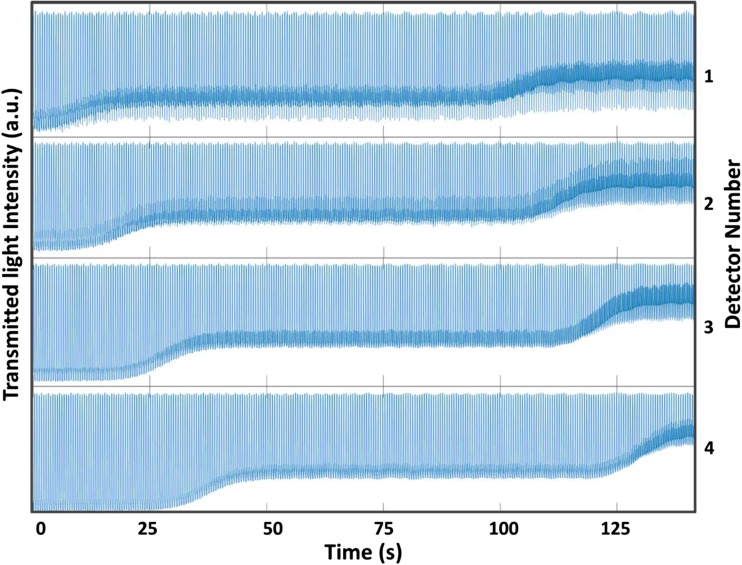
Calibration of the 4-detector flow cell via recording intensity change in 4 detectors (top to bottom) with changing dye concentrations (0.35, 0.25 and 0.15 mg/mL). It shows that the change in color intensity is preserved from top to bottom (detectors 1–4) and travels to next detectors without contamination or smearing

The reproducibility of the 4-detector flow cell was calibrated by generating droplets containing food dye and flowing them through the flow cell. Figure 3 shows the droplets of three different food dye concentrations (0.35, 0.25 and 0.15 mg/mL) passed through the flow cell, and the level of intensity at the top of the graph shows oil which was constant during the experiment. As the light passed through the droplet, the transmitted light intensity decreased as per Beer-Lambert law. The signal was then collected from the photodiode and plotted. The variation in intensity for each droplet at single concentration was found to be very low (%RSD < 0.5%).

Figure 3 indicates (from top to bottom) that the change in colour intensity of the droplets generated at T-junction is preserved without contamination or smearing along the tubing, and without dependence on length or flow rate of the droplets. In this experiment about 20 s were required to switch from one concentration to the other completely which can be further reduced by shortening the tubing lengths from the inlet reservoirs and the droplet generation.

**Fig. 4:**
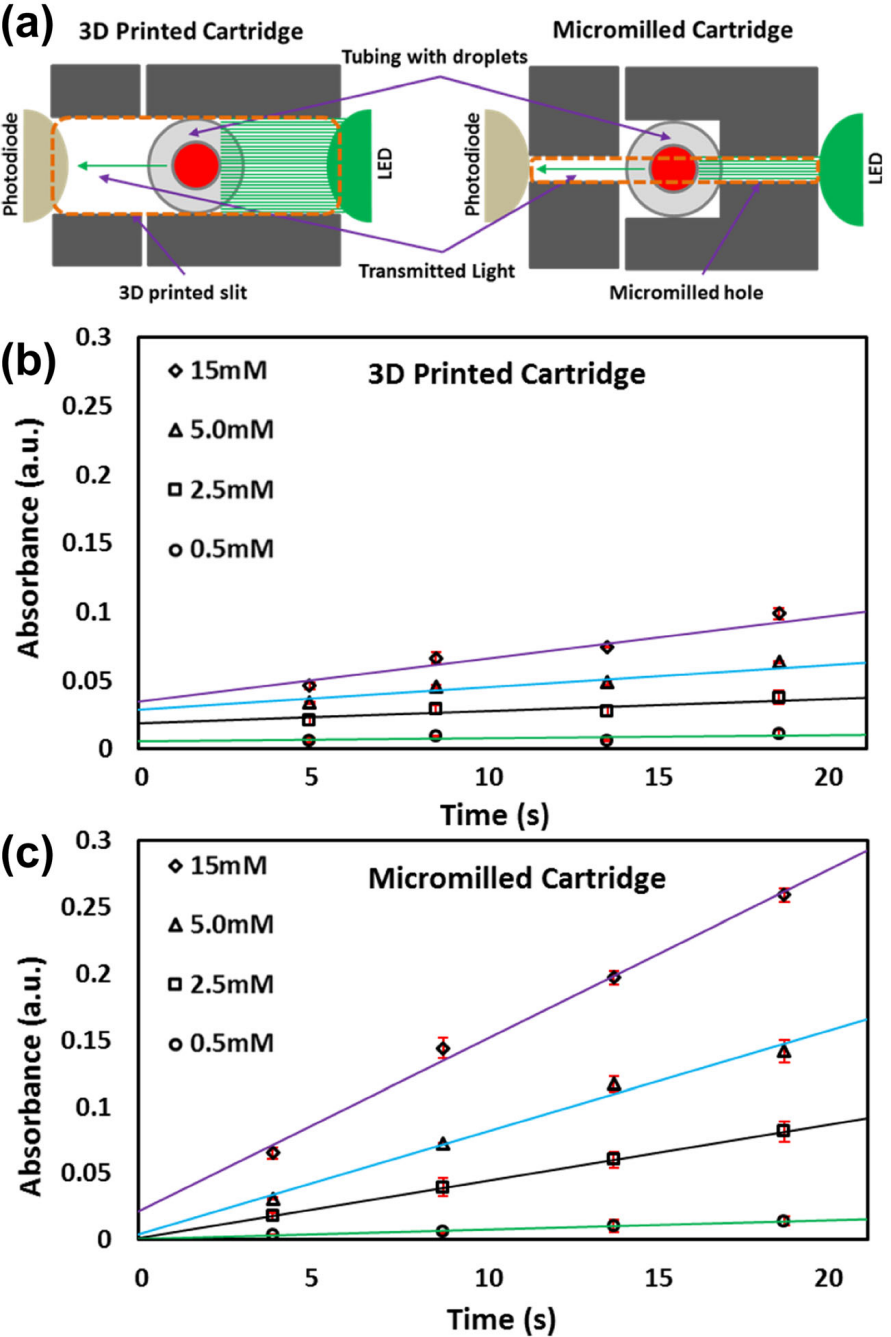
Comparison of the 3D printed and micromilled flow cell: **a** Schematic of the light absorbance via 3D printed (Hassan et al. 2016) and micromilled cartridge; **b** Absorbance measurements via 3D printed flow cell; **c** Absorbance measurements via micromilled flow cell. Glucose concentrations are 0.5, 2.5, 5, 15 mM

Figure 4a shows the schematic of the light absorption via 3D printed and micromilled cartridges. The dimensions of channels and grooves in the cartridge printed by 3D printer are less accurate and reproducible. These slight variations in dimensions allow misalignment of the holes and hence, more absorbance differences in between detectors. Furthermore, it can be seen from the schematic (Fig. 4a) that the light was passing through the whole tubing including the wall which affects the absorbance measurements (due to variations in refractive index). Small 3D printed holes (0.4 mm) were also printed and tested, however, the alignment was even more complex because slight variation in 3D printed dimensions including tubing holders can misalign the holes and reduce sensitivity. Therefore, micromilling was used to fabricate highly accurate sized holes in black PMMA pieces (Fig. 4a) and aligned using alignment pins. This method provided the best alignment of the holes and hence improved sensitivity of the flow cell (Fig. 4b and c).

Having calibrated the flow cell with glucose reaction droplets, a micromilled (4 detectors) flow cell and 3D printed flow cell (Hassan et al. 2016) were used to determine glucose concentrations. Contrary to the 7-detector flow cell reported previously (Hassan et al. 2016), 4-detector flow cell was found to be enough to achieve required resolution (R^2^ > 0.99). This is because to provide a flow cell with low-power consumption for POC applications. Droplets of different concentrations of glucose (0.5, 2.5, 5, 15 mM) were generated and flowed through the multi-detector flow cell. The droplets passed through the consecutive detectors and the absorbances were measured as shown in Fig. 4b and c. It can be clearly seen from the graphs that the absorbances were higher for the micromilled detector (similar concentrations). This is because of the reduced effect of refractive index due to the better alignment of the holes.

**Fig. 5:**
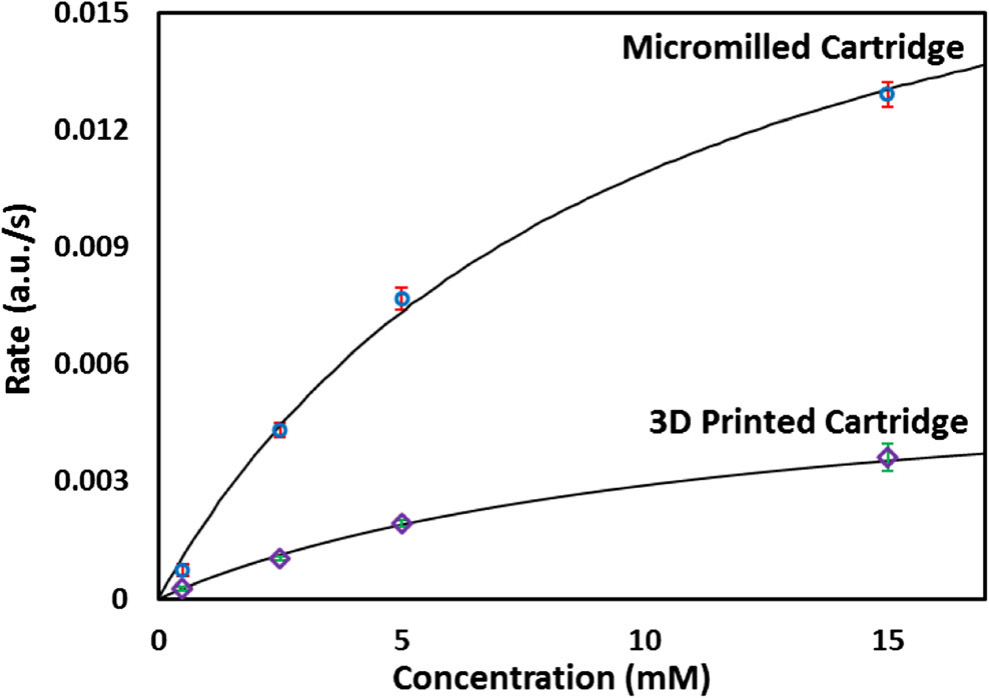
Comparison of the Michaelis-Menten curve for 3D printed and micromilled cartridge. The data is fitted by nonlinear regression with a Michaelis-Menten curve generating *Vmax* = 0.0060 a.u./s and *Km* = 11.48 mM (3D printed) and *Vmax* = 0.019 a.u./s and *Km* = 10.92 mM (micromilled)

Absorption increased linearly with the gradient of the line-of-best-fit giving the initial reaction rate (coloured quinoneimine). The limit of detection (LOD) was measured as three times the standard deviation (σ) of a series of blank measurements by slope of the calibration line [(3 x σ)/slope]. The LOD of the micromilled flow cell was found to be 0.1 mM as compared to 0.35 mM (3D-printed). In order to convert the measured reaction rates to a known concentration, the method must first be calibrated using the Michaelis-Menten model which relates the initial rate of reaction (*Vo*) to the concentration of the substrate. The initial rates of reaction were plotted against standard Michaelis-Menten fit as shown in Fig. 5. From the Michaelis-Menten, maximum rate at saturating substrate concentration known as *Vmax* and substrate concentration at half the maximum rate known as *Km* were calculated. The values of *Vmax* and *Km* were calculated to be *Vmax* = 0.0060 a.u./s and *Km* = 11.48 mM (3D printed) and *Vmax* = 0.019 a.u./s and *Km* = 10.92 mM (micromilled).

## Conclusions

This paper presents the development of optical flow cell for accurate quantification of enzymatic colorimetric assays in droplets. The flow cell was fabricated with precise micromilling, analogous to a previously reported 3D printed device (Hassan et al. 2016). The flow cell was capable of measuring absorbances with %RSD of 3.2% (detector-to-detector) as compared to 3D printed flow cell (6.5%). The flow cell is compact, low cost and can be used in portable analyzers.
